# 2-Eth­oxy-5-methylbis[1,2,4]triazolo[1,5-*a*:4′,3′-*c*]quinazoline

**DOI:** 10.1107/S1600536811052810

**Published:** 2011-12-14

**Authors:** Rashad Al-Salahi, Detlef Geffken, Ahmed Bari

**Affiliations:** a Department of Pharmaceutical Chemistry, College of Pharmacy, King Saud University, Riyadh 11451, Saudi Arabia; b Department of Chemistry, Institute of Pharmacy, University of Hamburg, Bundesstrasse 45, 20146, Hamburg, Germany

## Abstract

The title compound, C_13_H_12_N_6_O, is a functionalized ditriazoloquinazoline with substituted eth­oxy and methyl groups attached at the 2-position of each triazole spacer. The fused-ring system is essentially planar [r.m.s. deviation = 0.016 (2) Å]. In the crystal, a weak C—H⋯N hydrogen bond connects the mol­ecules into a chain along [101].

## Related literature

For the biological activity of quinazoline derviatives, see: Alagarsamy *et al.* (2005 ▶, 2007 ▶). For our study of the chemical and pharmacological properties of triazolo quinazoline deriv­atives, see: Al-Salahi (2009 ▶); Al-Salahi *et al.* (2010 ▶, 2011 ▶); Berezank *et al.* (2008*a* ▶,*b* ▶). For related structures, see: El-Tombary *et al.* (1999 ▶).
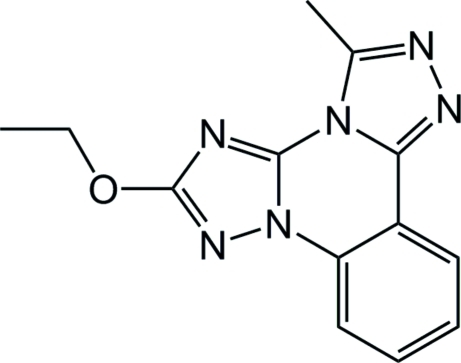


## Experimental

### 

#### Crystal data

C_13_H_12_N_6_O
*M* *_r_* = 268.29
Monoclinic, 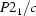

*a* = 7.4121 (11) Å
*b* = 19.240 (3) Å
*c* = 9.3096 (14) Å
β = 109.051 (2)°
*V* = 1254.9 (3) Å^3^
*Z* = 4
Mo *K*α radiation
μ = 0.10 mm^−1^
*T* = 153 K
0.43 × 0.21 × 0.05 mm

#### Data collection

Bruker SMART APEX CCD area-detector diffractometer
Absorption correction: multi-scan (*SADABS*; Bruker, 1998 ▶) *T*_min_ = 0.959, *T*_max_ = 0.995
2851 measured reflections
2851 independent reflections
1817 reflections with *I* > 2σ(*I*)
*R*_int_ = 0.053

#### Refinement

*R*[*F* ^2^ > 2σ(*F* ^2^)] = 0.055
*wR*(*F* ^2^) = 0.134
*S* = 0.91
2851 reflections
183 parameters
H-atom parameters constrained
Δρ_max_ = 0.29 e Å^−3^
Δρ_min_ = −0.24 e Å^−3^

### 

Data collection: *SMART* (Bruker, 1998 ▶); cell refinement: *SAINT* (Bruker, 1998 ▶); data reduction: *SAINT*; program(s) used to solve structure: *SHELXS97* (Sheldrick, 2008 ▶); program(s) used to refine structure: *SHELXL97* (Sheldrick, 2008 ▶); molecular graphics: *SHELXTL* (Sheldrick, 2008 ▶); software used to prepare material for publication: *publCIF* (Westrip, 2010 ▶).

## Supplementary Material

Crystal structure: contains datablock(s) I, global. DOI: 10.1107/S1600536811052810/is5002sup1.cif

Structure factors: contains datablock(s) I. DOI: 10.1107/S1600536811052810/is5002Isup2.hkl

Additional supplementary materials:  crystallographic information; 3D view; checkCIF report

## Figures and Tables

**Table 1:** Hydrogen-bond geometry (Å, °)

*D*—H⋯*A*	*D*—H	H⋯*A*	*D*⋯*A*	*D*—H⋯*A*
C3—H3⋯N4^i^	0.95	2.60	3.533 (2)	166

Symmetry code: (i) 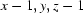.

**Fig. 1.**
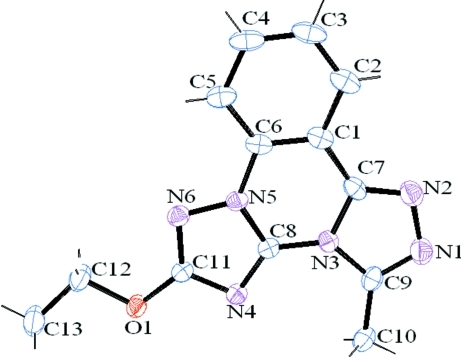
Fig. 1. The molecular structure of the title compound, showing 50% probability displacement ellipsoids for non-H atoms and the atom-numbering scheme.

## supplementary crystallographic information

### Comment

Recently, the triazolo-annelated quinazoline compounds were found to display remarkable pharmacological activities (Alagarsamy *et al.*, 2005, 2007). The novel 2-alkoxy-1,2,4-triazolo[1,5-*a*]quinazolines have been proven as excellent agents for controlling the plant growth diseases caused by fungal pathogen agents (Berezank *et al.*, 2008*a*,*b*), and described as adenosine receptor antagonists (Al-Salahi *et al.*, 2011). Furthermore, the bis-[1,2,4]triazolo[4,3 - a:4',3'-c]quinazoline has been reported to exhibit antitoxoplasmosis activity (El-Tombary *et al.*, 1999). This work has done in continuation of our program with the aim of obtaining an interesting series of bis-[1,2,4]triazoloquinazolines compounds which could be expected to contribute as a pharmacophore to the bioactivity of their triazoloquinazoline parent compounds (Al-Salahi *et al.*, 2009, 2010).

### Experimental

A mixture of 2-ethoxy-5-chloro-[1,2,4]triazolo[1,5-*a*]quinazoline (1 mmol) and acetohydrazide (2.2 mmol) was refluxed in absolute toluene (15 ml) in the presence of sodium hydride (0.8 mmol) for 18 h. The solvent was removed under reduced pressure, and the residue was crystallized from methanol to afford (C_13_H_12_N_6_O) as yellowish crystal.

### Refinement

All H atoms were positioned geometrically and refined using a riding model, with C—H = 0.95–0.98 Å. The displacement parameters are *U*_iso_(H) = *xU*_eq_(C), where *x* = 1.2 or 1.5.

### Crystal data

**Table:**

C_13_H_12_N_6_O	*F*(000) = 560
*M**_r_* = 268.29	*D*_x_ = 1.420 Mg m^−^^3^
Monoclinic, *P*2_1_/*c*	Mo *K*α radiation, λ = 0.71073 Å
Hall symbol: -P 2ybc	Cell parameters from 210 reflections
*a* = 7.4121 (11) Å	θ = 1.2–27.0°
*b* = 19.240 (3) Å	µ = 0.10 mm^−^^1^
*c* = 9.3096 (14) Å	*T* = 153 K
β = 109.051 (2)°	Plate, colourless
*V* = 1254.9 (3) Å^3^	0.43 × 0.21 × 0.05 mm
*Z* = 4	

### Data collection

**Table:**

Bruker SMART APEX CCD area-detector diffractometer	2851 independent reflections
Radiation source: fine-focus sealed tube	1817 reflections with *I* > 2σ(*I*)
graphite	*R*_int_ = 0.053
ω scan	θ_max_ = 27.5°, θ_min_ = 2.6°
Absorption correction: multi-scan (*SADABS*; Bruker, 1998)	*h* = −9→9
*T*_min_ = 0.959, *T*_max_ = 0.995	*k* = −24→24
2851 measured reflections	*l* = 0→11

### Refinement

**Table:**

Refinement on *F*^2^	Primary atom site location: structure-invariant direct methods
Least-squares matrix: full	Secondary atom site location: difference Fourier map
*R*[*F*^2^ > 2σ(*F*^2^)] = 0.055	Hydrogen site location: inferred from neighbouring sites
*wR*(*F*^2^) = 0.134	H-atom parameters constrained
*S* = 0.91	*w* = 1/[σ^2^(*F*_o_^2^) + (0.072*P*)^2^] where *P* = (*F*_o_^2^ + 2*F*_c_^2^)/3
2851 reflections	(Δ/σ)_max_ = 0.044
183 parameters	Δρ_max_ = 0.29 e Å^−^^3^
0 restraints	Δρ_min_ = −0.24 e Å^−^^3^

### Special details

**Table:**

Geometry. All e.s.d.'s (except the e.s.d. in the dihedral angle between two l.s. planes) are estimated using the full covariance matrix. The cell e.s.d.'s are taken into account individually in the estimation of e.s.d.'s in distances, angles and torsion angles; correlations between e.s.d.'s in cell parameters are only used when they are defined by crystal symmetry. An approximate (isotropic) treatment of cell e.s.d.'s is used for estimating e.s.d.'s involving l.s. planes.
Refinement. Refinement of *F*^2^ against ALL reflections. The weighted *R*-factor *wR* and goodness of fit *S* are based on *F*^2^, conventional *R*-factors *R* are based on *F*, with *F* set to zero for negative *F*^2^. The threshold expression of *F*^2^ > σ(*F*^2^) is used only for calculating *R*-factors(gt) *etc*. and is not relevant to the choice of reflections for refinement. *R*-factors based on *F*^2^ are statistically about twice as large as those based on *F*, and *R*- factors based on ALL data will be even larger.

### Fractional atomic coordinates and isotropic or equivalent isotropic displacement parameters (Å^2^)

**Table:**

	*x*	*y*	*z*	*U*_iso_*/*U*_eq_	
O1	0.26808 (18)	0.12586 (7)	0.60062 (14)	0.0292 (4)	
N5	−0.0311 (2)	0.21218 (8)	0.29706 (16)	0.0243 (4)	
N3	0.0101 (2)	0.33173 (8)	0.34526 (16)	0.0244 (4)	
N4	0.1812 (2)	0.23979 (8)	0.52010 (16)	0.0233 (4)	
N6	0.0364 (2)	0.14887 (8)	0.36339 (16)	0.0265 (4)	
N1	−0.0267 (3)	0.44495 (9)	0.32026 (18)	0.0375 (4)	
N2	−0.1545 (2)	0.41376 (9)	0.18987 (18)	0.0360 (4)	
C6	−0.1751 (3)	0.22080 (10)	0.1548 (2)	0.0263 (5)	
C1	−0.2270 (3)	0.28941 (10)	0.10826 (19)	0.0273 (5)	
C8	0.0587 (2)	0.26359 (10)	0.39243 (19)	0.0223 (4)	
C11	0.1609 (2)	0.16942 (10)	0.49439 (19)	0.0232 (4)	
C5	−0.2620 (3)	0.16363 (11)	0.0665 (2)	0.0322 (5)	
H5	−0.2268	0.1175	0.1003	0.039*	
C7	−0.1306 (3)	0.34608 (11)	0.20684 (19)	0.0276 (5)	
C4	−0.4021 (3)	0.17661 (12)	−0.0729 (2)	0.0379 (6)	
H4	−0.4620	0.1386	−0.1356	0.045*	
C3	−0.4562 (3)	0.24455 (12)	−0.1222 (2)	0.0379 (6)	
H3	−0.5523	0.2522	−0.2174	0.045*	
C9	0.0698 (3)	0.39608 (10)	0.4119 (2)	0.0292 (5)	
C2	−0.3704 (3)	0.30040 (12)	−0.0332 (2)	0.0349 (5)	
H2	−0.4079	0.3464	−0.0671	0.042*	
C10	0.2187 (3)	0.40648 (10)	0.5633 (2)	0.0336 (5)	
H10A	0.2326	0.4562	0.5868	0.050*	
H10B	0.3407	0.3877	0.5610	0.050*	
H10C	0.1805	0.3823	0.6414	0.050*	
C12	0.2394 (3)	0.05207 (10)	0.5604 (2)	0.0371 (5)	
H12A	0.2652	0.0432	0.4642	0.045*	
H12B	0.1059	0.0386	0.5465	0.045*	
C13	0.3737 (3)	0.01071 (11)	0.6867 (2)	0.0439 (6)	
H13A	0.5055	0.0238	0.6983	0.066*	
H13B	0.3556	−0.0389	0.6628	0.066*	
H13C	0.3479	0.0202	0.7816	0.066*	

### Atomic displacement parameters (Å^2^)

**Table:**

	*U*^11^	*U*^22^	*U*^33^	*U*^12^	*U*^13^	*U*^23^
O1	0.0254 (7)	0.0221 (8)	0.0340 (7)	0.0003 (6)	0.0013 (6)	−0.0025 (6)
N5	0.0207 (8)	0.0270 (10)	0.0242 (8)	−0.0019 (7)	0.0060 (6)	−0.0033 (7)
N3	0.0219 (8)	0.0257 (10)	0.0265 (8)	0.0020 (7)	0.0092 (7)	0.0015 (7)
N4	0.0174 (8)	0.0253 (10)	0.0263 (8)	−0.0008 (7)	0.0059 (7)	−0.0020 (7)
N6	0.0241 (8)	0.0264 (10)	0.0272 (8)	−0.0008 (7)	0.0061 (7)	−0.0031 (7)
N1	0.0416 (11)	0.0319 (11)	0.0399 (10)	0.0074 (9)	0.0147 (8)	0.0028 (8)
N2	0.0360 (10)	0.0366 (12)	0.0348 (9)	0.0086 (9)	0.0108 (8)	0.0047 (8)
C6	0.0185 (9)	0.0407 (13)	0.0216 (9)	−0.0026 (9)	0.0091 (7)	−0.0014 (9)
C1	0.0190 (9)	0.0404 (13)	0.0248 (9)	0.0005 (9)	0.0100 (8)	0.0021 (8)
C8	0.0169 (9)	0.0267 (11)	0.0249 (9)	−0.0012 (8)	0.0092 (8)	−0.0029 (8)
C11	0.0179 (9)	0.0252 (11)	0.0264 (9)	−0.0011 (8)	0.0073 (7)	−0.0038 (8)
C5	0.0256 (10)	0.0441 (14)	0.0282 (10)	−0.0102 (10)	0.0107 (8)	−0.0060 (9)
C7	0.0226 (10)	0.0359 (13)	0.0258 (9)	0.0068 (9)	0.0099 (8)	0.0062 (9)
C4	0.0254 (11)	0.0629 (17)	0.0263 (10)	−0.0151 (11)	0.0097 (9)	−0.0098 (10)
C3	0.0200 (10)	0.0670 (17)	0.0250 (10)	−0.0045 (11)	0.0050 (8)	0.0011 (10)
C9	0.0296 (11)	0.0266 (12)	0.0350 (10)	0.0017 (9)	0.0153 (9)	−0.0033 (9)
C2	0.0220 (10)	0.0553 (15)	0.0281 (10)	0.0034 (10)	0.0092 (9)	0.0076 (10)
C10	0.0338 (12)	0.0270 (12)	0.0394 (11)	−0.0031 (10)	0.0110 (9)	−0.0077 (9)
C12	0.0357 (12)	0.0240 (12)	0.0473 (12)	−0.0033 (10)	0.0076 (10)	−0.0056 (10)
C13	0.0462 (14)	0.0250 (13)	0.0560 (14)	0.0026 (10)	0.0106 (11)	0.0014 (10)

### Geometric parameters (Å, °)

**Table:**

O1—C11	1.341 (2)	C5—C4	1.395 (3)
O1—C12	1.466 (2)	C5—H5	0.9500
N5—C8	1.351 (2)	C4—C3	1.400 (3)
N5—N6	1.383 (2)	C4—H4	0.9500
N5—C6	1.414 (2)	C3—C2	1.379 (3)
N3—C9	1.391 (2)	C3—H3	0.9500
N3—C8	1.392 (2)	C9—C10	1.493 (3)
N3—C7	1.395 (2)	C2—H2	0.9500
N4—C8	1.320 (2)	C10—H10A	0.9800
N4—C11	1.375 (2)	C10—H10B	0.9800
N6—C11	1.327 (2)	C10—H10C	0.9800
N1—C9	1.313 (2)	C12—C13	1.497 (3)
N1—N2	1.407 (2)	C12—H12A	0.9900
N2—C7	1.317 (3)	C12—H12B	0.9900
C6—C5	1.398 (3)	C13—H13A	0.9800
C6—C1	1.403 (3)	C13—H13B	0.9800
C1—C2	1.412 (3)	C13—H13C	0.9800
C1—C7	1.454 (3)		
			
C11—O1—C12	114.54 (14)	C5—C4—H4	119.4
C8—N5—N6	108.83 (14)	C3—C4—H4	119.4
C8—N5—C6	126.11 (16)	C2—C3—C4	120.26 (19)
N6—N5—C6	125.03 (15)	C2—C3—H3	119.9
C9—N3—C8	133.29 (16)	C4—C3—H3	119.9
C9—N3—C7	105.59 (16)	N1—C9—N3	108.74 (17)
C8—N3—C7	121.08 (16)	N1—C9—C10	126.52 (18)
C8—N4—C11	100.42 (14)	N3—C9—C10	124.74 (17)
C11—N6—N5	100.92 (14)	C3—C2—C1	120.2 (2)
C9—N1—N2	108.99 (16)	C3—C2—H2	119.9
C7—N2—N1	106.98 (15)	C1—C2—H2	119.9
C5—C6—C1	122.08 (18)	C9—C10—H10A	109.5
C5—C6—N5	121.37 (18)	C9—C10—H10B	109.5
C1—C6—N5	116.54 (16)	H10A—C10—H10B	109.5
C6—C1—C2	118.41 (18)	C9—C10—H10C	109.5
C6—C1—C7	118.80 (16)	H10A—C10—H10C	109.5
C2—C1—C7	122.79 (19)	H10B—C10—H10C	109.5
N4—C8—N5	112.61 (16)	O1—C12—C13	108.09 (16)
N4—C8—N3	129.94 (16)	O1—C12—H12A	110.1
N5—C8—N3	117.45 (16)	C13—C12—H12A	110.1
N6—C11—O1	123.95 (17)	O1—C12—H12B	110.1
N6—C11—N4	117.22 (16)	C13—C12—H12B	110.1
O1—C11—N4	118.83 (15)	H12A—C12—H12B	108.4
C4—C5—C6	117.8 (2)	C12—C13—H13A	109.5
C4—C5—H5	121.1	C12—C13—H13B	109.5
C6—C5—H5	121.1	H13A—C13—H13B	109.5
N2—C7—N3	109.70 (17)	C12—C13—H13C	109.5
N2—C7—C1	130.31 (17)	H13A—C13—H13C	109.5
N3—C7—C1	119.98 (17)	H13B—C13—H13C	109.5
C5—C4—C3	121.25 (19)		
			
C8—N5—N6—C11	0.41 (17)	C8—N4—C11—O1	178.70 (15)
C6—N5—N6—C11	−177.68 (15)	C1—C6—C5—C4	1.1 (3)
C9—N1—N2—C7	−0.1 (2)	N5—C6—C5—C4	−179.63 (15)
C8—N5—C6—C5	−178.46 (16)	N1—N2—C7—N3	−0.09 (19)
N6—N5—C6—C5	−0.7 (3)	N1—N2—C7—C1	178.94 (18)
C8—N5—C6—C1	0.9 (2)	C9—N3—C7—N2	0.26 (19)
N6—N5—C6—C1	178.64 (15)	C8—N3—C7—N2	178.22 (14)
C5—C6—C1—C2	−0.6 (3)	C9—N3—C7—C1	−178.89 (15)
N5—C6—C1—C2	−179.99 (15)	C8—N3—C7—C1	−0.9 (2)
C5—C6—C1—C7	179.69 (16)	C6—C1—C7—N2	−179.24 (17)
N5—C6—C1—C7	0.3 (2)	C2—C1—C7—N2	1.1 (3)
C11—N4—C8—N5	0.72 (18)	C6—C1—C7—N3	−0.3 (2)
C11—N4—C8—N3	180.00 (16)	C2—C1—C7—N3	−179.94 (15)
N6—N5—C8—N4	−0.8 (2)	C6—C5—C4—C3	−0.9 (3)
C6—N5—C8—N4	177.30 (15)	C5—C4—C3—C2	0.2 (3)
N6—N5—C8—N3	179.86 (13)	N2—N1—C9—N3	0.3 (2)
C6—N5—C8—N3	−2.1 (2)	N2—N1—C9—C10	−179.39 (17)
C9—N3—C8—N4	0.1 (3)	C8—N3—C9—N1	−177.93 (16)
C7—N3—C8—N4	−177.21 (17)	C7—N3—C9—N1	−0.3 (2)
C9—N3—C8—N5	179.34 (16)	C8—N3—C9—C10	1.8 (3)
C7—N3—C8—N5	2.0 (2)	C7—N3—C9—C10	179.35 (16)
N5—N6—C11—O1	−179.07 (15)	C4—C3—C2—C1	0.2 (3)
N5—N6—C11—N4	0.04 (19)	C6—C1—C2—C3	0.0 (3)
C12—O1—C11—N6	0.7 (2)	C7—C1—C2—C3	179.65 (17)
C12—O1—C11—N4	−178.38 (14)	C11—O1—C12—C13	177.76 (15)
C8—N4—C11—N6	−0.47 (19)		

### Hydrogen-bond geometry (Å, °)

**Table:**

*D*—H···*A*	*D*—H	H···*A*	*D*···*A*	*D*—H···*A*
C3—H3···N4^i^	0.95	2.60	3.533 (2)	166

Symmetry codes: (i) *x*−1, *y*, *z*−1.
